# Efficacy and safety of bismuth quadruple regimens containing minocycline and vonoprazan for eradication of *Helicobacter pylori*: Real‐world evidence

**DOI:** 10.1002/jgh3.13070

**Published:** 2024-05-01

**Authors:** Qiyunna He, Yan Ou, Huili Zhu, Zhiqian Chen, Dailan Yang, Qian Cheng, Xia Yin, Lina Xiao, Lin Cai, Yan Ye, Xin Xu, Juan Liao

**Affiliations:** ^1^ West China School of Public Health and West China Fourth Hospital Sichuan University Chengdu Sichuan China; ^2^ Department of Gastroenterology, West China School of Public Health and West China Fourth Hospital Sichuan University Chengdu China; ^3^ Non‐Communicable Diseases Research Center, West China‐PUMC C.C. Chen Institute of Health Sichuan University Chengdu China

**Keywords:** efficacy, eradication, *Helicobacter pylori*, minocycline, vonoprazan

## Abstract

**Background and Aim:**

To evaluate the efficacy and safety of minocycline, vonoprazan, amoxicillin, and bismuth quadruple therapy for *Helicobacter pylori* (*H. pylori*) treatment.

**Methods:**

From August 2022 to May 2023, clinical data were collected from patients who received *H. pylori* eradication treatment at West China Fourth Hospital, Sichuan University. One group received the MVAB regimen (amoxicillin, minocycline, vonoprazan, and colloidal bismuth pectin), while another group received the FOAB regimen (amoxicillin, furazolidone, omeprazole, and colloidal bismuth pectin), both administered for 14 days. Follow‐up assessments of safety and compliance were conducted within 1 week after treatment completion. One and a half months after treatment, the success of eradication was evaluated using the urea breath test.

**Results:**

For the MVAB regimen as a first‐line treatment, the eradication rate was 90.1% (127/141, 95% CI: 85.1–95.1%) in the ITT analysis and 93.4% (127/136, 95% CI: 89.2–97.6%) in the PP analysis as a first‐line treatment. As a second‐line treatment, the eradication rate was 91.3% (21/23, 95% CI: 78.8–103.8%) in both analyses. For the FOAB regimen as a first‐line treatment, the eradication rate was 98.0% (50/51, 95% CI: 94.1–101.2%) in the ITT analysis and 100% (50/50, 95% CI: 100%) in the PP analysis. As a second‐line treatment, the eradication rate was 100% (6/6, 95% CI: 100%) in both analyses. Moreover, there was no significant difference in the incidence of adverse events between the two groups (MVAB regimen: 5.5% and FOAB regimen: 8.8%; *P* > 0.05).

**Conclusions:**

The MVAB regimen could indeed be a viable alternative treatment option to conventional therapies.

## Graphical abstract

The combination of amoxicillin, minocycline, vonoprazan, and colloidal bismuth pectin regimen could certainly serve as a viable alternative treatment option compared to conventional therapies.

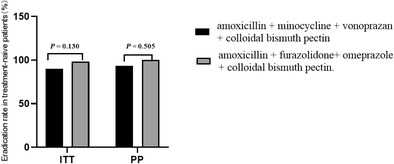

## Introduction

Due to factors such as antibiotic misuse, inappropriate treatment regimens, and poor patient compliance, the efficacy of the commonly used triple therapy for eradicating *Helicobacter pylori* (*H. pylori*) has significantly declined,^1^, ^2^ especially with the emergence of high antibiotic resistance, which severely affects the eradication rate of *H. pylori*.^3^, ^4^, ^5^ Additionally, *H. pylori* can be multi‐resistant to antibiotics.^6^ Consequently, successfully eradicating *H. pylori* infection has become a global challenge and an urgent problem.

The sixth Chinese National Consensus Report on the management of *H. pylori* infection recommends quadruple therapy for eradicating *H. pylori*, which includes bismuth, PPIs, and two antibiotics. PPIs are frequently used to control intragastric Ph levels by reducing acid secretion. However, the effectiveness of PPIs in suppressing acid production may be influenced by drug doses and the host's CYP2C19 gene polymorphism,^4^ which can impact the outcomes of *H. pylori* eradication regimens containing PPIs. Vonoprazan fumarate, a novel potassium‐competitive acid blocker (P‐CAB), has been proven to be more effective in suppressing acid production compared with PPIs, with faster onset, longer duration of action, and better outcomes. It has been used clinically for approximately 7 years, particularly in Asian countries. The prevalence of *H. pylori* resistance to metronidazole and clarithromycin, two antibiotics frequently utilized in *H. pylori* eradication treatments, has been increasing in China. Conversely, tetracycline, another antibiotic option, has a low reported resistance rate of just 2%,^5^ making it a valuable first‐line choice for *H. pylori* eradication in China. Nevertheless, it can be difficult to obtain tetracycline for clinical use in certain regions, and its common adverse effects, including gastrointestinal symptoms, pigmentation changes, and central nervous system effects, may seriously affect patient compliance and quality of life.^7^ As a result, the limited accessibility and potential side effects of tetracycline may impede its use. Minocycline is a type of semi‐synthetic tetracycline with fewer side effects and better bactericidal activity than tetracycline. Due to its long half‐life, it can be taken once or twice a day, which can improve patient compliance. Furthermore, its high lipophilicity and small weight result in higher absorption rates.^8^, ^9^ Previous studies have reported that minocycline can effectively eradicate *H. pylori* in vitro with a resistance rate of only 6.6%.^10^ Therefore, the use of vonoprazan in combination with minocycline‐containing regimens may provide a promising treatment option for *H. pylori* eradication, especially in regions with high antibiotic resistance.

## Methods

### Study design and participants

This is a real‐world study conducted in the Digestive Department of West China Fourth Hospital, Sichuan University. Clinical data were collected from patients undergoing *H. pylori* eradication treatment with the minocycline‐vonoprazan quadruple regimen and the furazolidone‐omeprazole quadruple regimen between August 2022 and May 2023. These patients included both those undergoing treatment for the first time and those with a history of previous *H. pylori* eradication.

### Diagnosis of *H. pylori* and treatment regimen

Both positive diagnosis and eradication diagnosis of *H. pylori* used ^13^C‐UBT or ^14^C‐UBT. The MVAB regimen consisted of vonoprazan (VPZ, 20 mg/tablet, Takeda Pharmaceutical Co.) 20 mg once daily (half an hour before breakfast/lunch and dinner), minocycline 100 mg twice daily (half an hour after breakfast/lunch and dinner), amoxicillin 1000 mg twice daily (half an hour after breakfast/lunch and dinner), and colloidal bismuth pectin 300 mg twice daily (half an hour before breakfast/lunch and dinner) for 14 days. The FOAB regimen consisted of furazolidone 100 mg twice daily (half an hour after breakfast/lunch and dinner), omeprazole 20 mg twice daily (half an hour before breakfast/lunch and dinner), amoxicillin 1000 mg twice daily (half an hour after breakfast/lunch and dinner), and colloidal bismuth pectin 300 mg twice daily (half an hour before breakfast/lunch and dinner). Both regimens were administered for 14 days.

### Study outcomes

The main objective of this study was to determine the success rate of eliminating *H. pylori*, which was evaluated using either ^13^C‐urea breath test or ^14^C‐urea breath test at least one and a half months after completing the therapy. The ^13^C‐urea breath test cutoff value was 4.0‰ (delta over baseline, DOB), while the ^14^C‐urea breath test cutoff value was 100 (disintegrations per minute, DPM). Prior to testing, patients were required to fast. To ensure accurate results, patients were prohibited from taking antibiotics, PPIs, or P‐CAB between the end of treatment and the urea breath test follow‐up examination.

The study also assessed secondary endpoints, such as the occurrence of adverse reactions, patient compliance, and other factors that may impact the treatment's effectiveness. Patients were followed up via telephone within 1 week after completing the medication to obtain information on adverse reactions and compliance.

### Statistical analysis

Collected data were analyzed using IBM SPSS Statistics 26.0 statistical software (IBM Co.). Continuous variables were expressed as the mean ± SD. Categorical variables were presented as frequencies. The eradication rate of *H. pylori* was calculated using intention‐to‐treat (ITT) and per‐protocol (PP) analyses, with a 95% confidence interval (CI) also calculated. Intergroup comparisons were conducted using either the chi‐squared test or Fisher's exact probability test. The factors influencing successful eradication were analyzed using logistic regression. Statistical significance was defined as *P* < 0.05.

## Results

### Patient demographics and clinical characteristics

The study included a total of 221 patients, with 112 males and 109 females, and an average age of 44.51 ± 13.378 years. Between the two groups of patients, there were no differences in terms of gender, symptoms before treatment, alcohol consumption history, endoscopic results, and previous history of eradication treatment (*P* > 0.05). However, there were differences in smoking and alcohol consumption (*P* < 0.05). The detailed baseline demographic data are presented in Table 1.

**Table 1:** Demographic and clinical data of all patients

	The MVAB regimen *n* = 164	The FOAB regimen *n* = 57	*P* value
Male	81 (49.4%)	31 (54.4%)	0.516
Age	43.77	46.63	‐
Clinical symptom			
Abdominal discomfort	17 (10.4%)	8 (14.0%)	0.45
Bad breath	5 (3.0%)	2 (3.5%)	1
Acid reflux	5 (3.0%)	1 (1.8%)	1
Belching	2 (1.2%)	1 (1.8%)	1
Nausea	2 (1.2%)	1 (1.8%)	1
Endoscopy diagnosis before treatment			
Chronic gastritis	50 (30.4%)	18 (32.6%)	0.878
Gastric polyp	5 (3.0%)	2 (3.5%)	1
Gastroesophageal reflux	5 (3.0%)	1 (1.8%)	1
Gastric ulcer	3 (1.8%)	1 (1.8%)	1
Smoking	8 (4.9%)	10 (17.5%)	0.003
Drinking	13 (7.9%)	10 (17.5%)	0.041
Received eradication therapy	23 (14.0%)	6 (10.5%)	0.500

### *H. pylori* eradication rates

A total of 164 patients received the MVAB regimen, with 148 patients successfully cured, 11 experiencing treatment failure, and 5 discontinued voluntarily treatment due to adverse reactions. Among these patients, 141 were receiving eradication therapy for the first time, while 23 had received eradication therapy previously. For patients with first‐time therapy, the eradication rate of 90.1% (127/141, 95% CI 85.1–95.1%) was based on ITT analysis, and 93.4% (127/136, 95% CI 89.2–97.6%) was based on PP analysis. For patients with previous therapy, the eradication rate was 91.3% (21/23, 95% CI 89.2–97.6%) based on both ITT and PP analyses.

**Figure 1:**
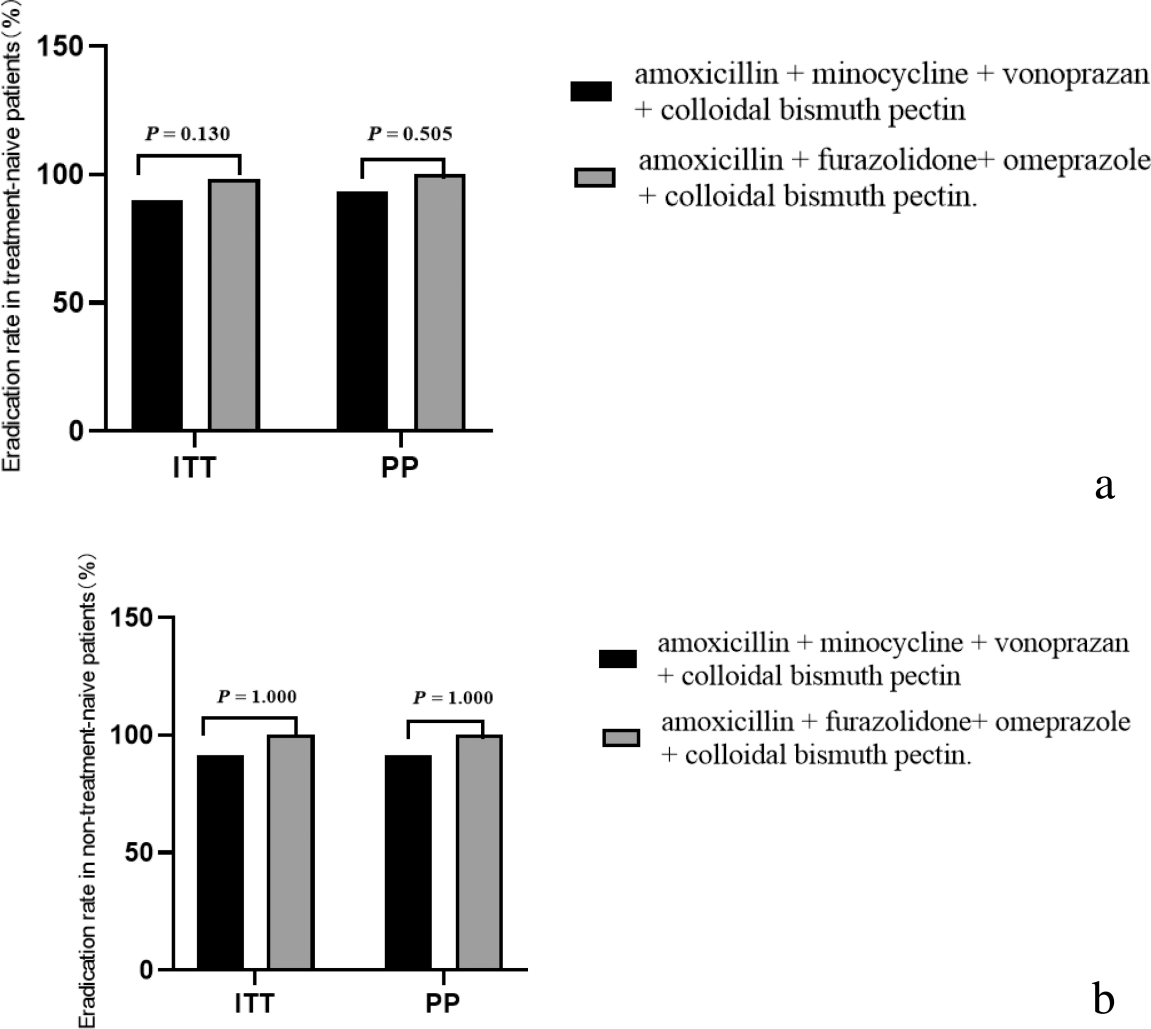
Success rate of two eradication therapies. ITT, intention‐to‐treat; PP, per‐protocol. (a) The eradication rate for first‐time treatment patient for both regimens. (b) The eradication rate for patients who had received eradication therapy previously for both regimens.

For the FOAB regimen, of 57 enrolled patients, 56 were successfully cured, with 1 patient discontinuing medication for reasons unrelated to adverse reactions. Among these patients, 51 were receiving eradication therapy for the first time and 6 had received it previously. For patients taking the therapy for the first time, the eradication rate of this regimen was 98.0% (50/51, 95% CI 94.1–101.2%) based on ITT analysis, and 100% (50/50, 95% CI 100%) based on PP analysis. For patients with previous therapy, the eradication rate of this regimen was 100% (6/6, 95% CI 100%), according to both ITT and PP analyses. The success rates of both regimens are illustrated in Figure 1.

### Compliance and adverse reactions

Based on the MVAB regimen group, the compliance rate was 96.95% (159/164), and for the FOAB regimen group, it was 98.2% (56/57). There was no statistically significant difference in compliance between the two groups (*P* > 0.05). In MVAB regimen group, the adverse events included abdominal discomfort (1.2%, 2/164), nausea (1.2%, 2/164), diarrhea (0.61%, 1/164), dizziness (0.61%, 1/164), ataxia (0.61%, 1/164), soft stools (0.61%, 1/164), and gingival swelling and pain (0.61%, 1/164). Among the nine patients who experienced adverse reactions, one patient developed diarrhea two days after taking the medication and voluntarily discontinued treatment. Two patients experienced abdominal discomfort on the fifth and sixth days of medication respectively and voluntarily discontinued treatment. One patient experienced dizziness after 5 days of medication and voluntarily discontinued treatment. Another patient developed ataxia after 3 days of medication and voluntarily discontinued treatment. For the FOAB regimen group, adverse events included nausea (3.51%, 2/57), indigestion (3.51%, 2/57), and bloating (1.75%, 1/57). Additionally, there were no significant differences in adverse events between the two groups (*P* > 0.05), as shown in Table 2.

**Table 2:** Adherence and adverse events of the two eradication therapy

Variable	Minocycline‐vonoprazan quadruple therapy *n* = 164	Furazolidone‐omeprazole quadruple therapy *n* = 57	*P* value
Adherence	159 (96.95%)	56 (98.2%)	0.301
Adverse event			0.553
Abdominal discomfort	2	0	1
Nausea	2	2	0.274
Diarrhea	1	0	0.45
Dizziness	1	0	0.45
Ataxia	1	0	0.45
Soft stools	1	0	0.45
Gingival swelling and pain	1	0	0.45
Indigestion	0	2	0.066
Bloating	0	1	‐

### Factors affecting eradication rates

The factors related to the failure of *H. pylori* eradication therapy are shown in Table 3. In our study, patient gender, age, prior history of receiving eradication therapy, smoking, and alcohol consumption were not associated with eradication failure (*P* > 0.05).

**Table 3:** The factors influencing the success of *H. pylori* eradication

Factors	*P*	OR	95% CI
Gender	0.846	0.906	0.336–2.442
Years (<45)	0.876	1.082	0.401–2.915
Received eradication therapy	0.863	1.144	0.248–5.283
Smoking	0.9846	0.906	0.336–2.442
Drinking	0.953	1.047	0.226–4.855

## Discussion

We conducted a real‐world study using minocycline instead of tetracycline and vonoprazan instead of PPIs. The study aimed to evaluate the effectiveness and safety of a quadruple therapy regimen comprising amoxicillin, minocycline, vonoprazan, and colloidal bismuth pectin in eradicating *H. pylori* infection. In this study, we included patients who were receiving *H. pylori* eradication therapy for the first time or had received eradication therapy previously. For initial treatment patients, the eradication rate of *H. pylori* with MVAB regimen was 90.1% in the ITT analysis and 93.4% in the PP analysis. For nontreatment‐naive patients, the eradication rate of this therapy was 91.3% in both the ITT and PP analyses. There was no statistically significant difference in the eradication rates compared with FOAB regimen. Given that acceptable regimens typically require a >85% eradication rate in ITT analysis and >90% eradication rate in PP analysis.^11^ MVAB regimen achieved acceptable eradication rates.

In the study by Song *et al*.,^12^ the eradication rate of the esomeprazole, minocycline, metronidazole, and bismuth combination therapy as first‐line treatment was 85.5% (95% CI 79.6–91.4%) in the ITT analysis and 92.6% (95% CI 88.1–96.3%) in the PP analysis. As second‐line treatment, the eradication rate was 82.8% (95% CI 71.9–90.6%) in the ITT analysis and 89.5% (95% CI 80.7–96.5%) in the PP analysis. Another study by Song *et al*.^13^ showed that the eradication rate of the lansoprazole, minocycline, amoxicillin, and bismuth combination therapy as first‐line treatment was 87.5% (95% CI 81.9–92.5%) in the ITT analysis and 92.6% (95% CI 88.5–96.6%) in the PP analysis. For second‐line treatment, the eradication rates were 82.9% (95% CI 74.3–91.4%) and 89.1% (95% CI 81.3–95.3%), respectively. Our study results show that the eradication rates of the MVAB regimen in both ITT and PP analyses are higher than the aforementioned two regimens. This could be attributed to the rapid and potent acid‐suppressive effects of vonoprazan and the high sensitivity of *H. pylori* to amoxicillin.

In the study by Gu *et al*.,^14^ when the combination therapy of bismuth, rabeprazole, amoxicillin, and levofloxacin was used as first‐line treatment, the eradication rate was 89.8% (95% CI 84.3–95.4%) in the ITT analysis and 93.8% (95% CI 89.2–98.3%) in the PP analysis. Our study results show that the eradication rates of the MVAB regimen in both ITT and PP analyses are higher. A study on antibiotic resistance in various regions conducted by the World Health Organization showed that the global resistance rate to minocycline is less than 10%.^15^ This indicates that quadruple therapy including minocycline is an advantageous regimen with high eradication rates and low resistance rates.

Minocycline may cause neurologically related side effects due to its ability to penetrate the blood–brain barrier more easily than other tetracyclines. In a multicenter, randomized controlled study conducted by Huang *et al*.,^16^ the efficacy of minocycline and tetracycline in bismuth‐containing quadruple therapy for salvage treatment of *H. pylori* infection was compared. The results showed that there was no statistically significant difference in the incidence of adverse events between the minocycline group and the tetracycline group, with both showing relatively high rates (55.4% [102/184] and 53.3% [98/184], *P* > 0.05). But a meta‐analysis of randomized controlled trials on minocycline quadruple therapy for eradicating *H. pylori* ^17^ showed that the incidence of adverse reactions in the minocycline quadruple therapy group was similar to that in comparator groups using doxycycline, tetracycline, clarithromycin, and others. The results of our study showed that the common adverse reactions associated with the minocycline‐vonoprazan regimen were abdominal discomfort and nausea. Additionally, the incidence of adverse reactions was low (5.5%, 9/164), with the majority being mild to moderate. A very small number of patients (3.0%, 5/164) discontinued treatment due to intolerable adverse reactions. Furthermore, no serious adverse events occurred.

Our study demonstrated that minocycline‐vonoprazan quadruple therapy, nocycline, has a promising safety, with satisfactory eradication efficacy as a potential alternative treatment option for *H. pylori* infection.

Despite these promising results, our study has some limitation. This was a real‐world study and not a randomized controlled trial, and the follow‐up time was short, with insufficient information on baseline characteristics and symptom relief after eradication. Additionally, due to the small sample size, statistically significant results were not obtained. Therefore, future multicenter randomized controlled trials with larger sample sizes, more comprehensive baseline data, and longer follow‐up periods are needed to confirm the efficacy and safety of this quadruple therapy. These studies can also help determine the optimal dosage and duration of treatment and compare the efficacy and safety of this therapy with other commonly used treatment options.

## Conclusion

The quadruple therapy consisting of amoxicillin, minocycline, vonoprazan, and colloidal bismuth pectin is effective and safe for eradicating *H. pylori*. Additionally, the patient compliance is high.

## Patient consent statement

This study used standard second‐line medication, so there was no patient consent statement.

## Ethics statement

This is a real‐world study. Therefore, the paper is exempt from further Ethical Committee approval.

## Data Availability

All data underlying the results are available as part of the article, and no additional source data are required.
